# Cultural Values and the Coliform Bacterial Load of “*Masato*,” an Amazon Indigenous Beverage

**DOI:** 10.1007/s10393-020-01498-5

**Published:** 2020-11-20

**Authors:** Alejandra Bussalleu, Aldo Di-Liberto, Cesar Carcamo, Gabriel Carrasco-Escobar, Carol Zavaleta-Cortijo, Matthew King, Lea Berrang-Ford, Dora Maurtua, Alejandro Llanos-Cuentas, Indigenous Health and Adaption to Climate Change Research Group (IHACC), Patricia Garcia, Sherilee L. Harper, Victoria Edge, James Ford, Shuaib Lwasa, Didacus B. Namanya

**Affiliations:** 1 Facultad de Salud Pública y Administración, Universidad Peruana Cayetano Heredia, Av. Honorio Delgado 430, Lima 31, Peru; 2 Department of Population Medicine, University of Guelph, 50 Stone Road East, Guelph, ON N1G 2W1 Canada; 3 Geography Department, McGill University, Burnside Hall Building, Room 705, 805 Sherbrooke Street West, Montreal, QC H3A0B9 Canada; 4 Priestley International Centre for Climate, University of Leeds, Woodhouse Lane, Leeds, LS2 9JT UK; 5 Laboratorio de Bacteriología de los Laboratorios de Investigación y Desarrollo, Facultad de Ciencias y Filosofía, Universidad Peruana Cayetano Heredia, Av. Honorio Delgado 430, Lima 31, Peru; 6 Present Address: Division of Infectious Diseases, Department of Medicine, University of California San Diego, La Jolla, CA USA; 7 Present Address: Facultad de Salud Pública y Administración, Universidad Peruana Cayetano Heredia, Av. Honorio Delgado 430, Lima 31, Peru

**Keywords:** Water safety, Fermentation, Indigenous food, Amazonia, Shawi, Peru, *Masato*, Indigenous knowledge

## Abstract

Access to safe drinking water is limited in many isolated areas, such as the Amazon where Indigenous peoples frequently reside. Identifying safe forms of drinking water accepted by the communities could have positive health benefits for Indigenous peoples. Many Amazon Indigenous peoples traditionally prepare and consume a fermented beverage called *masato,* which is frequently the only form of water consumption. Despite its widespread consumption and evidence of the health benefits of fermentation, *masato* remains poorly investigated. We partnered with a Shawi Indigenous community in the Peruvian Amazon to conduct participatory photography to research *masato* preparation, and to characterize key cultural features and to assess the presence of total and fecal coliform bacteria by using a membrane filter technique. Pictures show that *masato* preparation is a key part of cultural practices and that there are clear gender roles in the preparation process. We found that 100% of communal water sources (26/26) were contaminated with coliform bacteria; by contrast, fewer, 18% of masato samples (2/11), were positive for coliform. This exploratory study suggests that fermented beverages like masato merit further investigation as they represent an Indigenous method to improve water quality in Amazonian communities where water safety cannot be assured.

## Introduction

Access to safe drinking water is essential for human health. Though estimates indicate drinking water is accessible to over three quarters of the global population, access to clean water and sanitation are far from universal in most low- and middle-income countries (Guardiola et al. 2010). Access to safe drinking water is especially limited for Indigenous populations located in the Amazon (Miranda et al. 2010). Indigenous communities in the Amazon often draw their water directly from rivers, streams, and lakes (Leite et al. 2013; McClain et al. 2001). Microbiological (i.e., bacterial, parasitic, and viral pathogens) and chemical (e.g., chemicals related to oil extraction) contaminants in untreated water present a risk to human health. Even in communities where water treatment campaigns have been implemented, researchers have found that less than half of the population in these Indigenous communities report boiling or treating water with chlorine before consumption (Brierley et al. 2014; Hofmeijer et al. 2013; INEI 2008; Nawaz et al. 2001). The lack of adoption of water treatment highlights the need to identify and understand Indigenous values and motivations associated with drinking water, and potential local alternatives for providing safe and culturally acceptable drinking water. Improved access to safe drinking water in the Amazon region could lead to positive health impacts for Indigenous peoples.

Across the Amazon region, there is a common practice among Indigenous communities of preparing and drinking a beverage called *masato* (Freire et al. 2016; Sedano 2006). *Masato* is the primary, and sometimes exclusive, form of water consumption (Torres-Slimming et al. 2019). It is a fermented beverage that has a low alcohol content and a slightly sweet and acidic taste (Sedano 2006). Women are usually responsible for the preparation of *masato* via boiling and mashing cassava, which is commonly known as “*yuca*” in Spanish and “*kisha*” in the Shawi language (Colehour et al. 2014; Sedano 2006; Zavaleta et al. 2017). *Masato* is also a culturally important beverage; for instance, research has shown that *masato* is widely consumed and culturally acceptable among Shawi communities in Peru (Hofmeijer et al. 2013; Ormaeche Macassi 2008; Zavaleta et al. 2018). Women masticate (chew) pieces of boiled cassava and periodically spit them into a mash. By consequence, in addition to the normal environmental community of microbes, human microbiota are also incorporated into the beverage during the fermentation process (Colehour et al. 2014).

Despite the potential health benefits of fermentation and the widespread consumption of *masato* in many Amazon Indigenous communities, neither the fermentation process per se, nor the microbiological characteristics of *masato* have been researched thoroughly (Freire et al. 2016). Studies suggest that the use of fermentation techniques has beneficial protective properties as the process may protect against microbial contamination with harmful pathogens, thus reducing risk of diarrheal disease (Marco et al. 2017; Şanlier et al. 2019; Watson et al. 1996). Diarrheal illness is a public health concern, particularly among children and Indigenous populations in the Peruvian Amazon. According to a recent study, the prevalence of reported acute diarrheal events using a 15-day recall period was 19% among children in the Loreto region (Perú 2018), increasing to 50% in Indigenous children in the region of Amazonas in northern Peru (Diaz et al. 2015). A better characterization of cultural importance of *masato* for Indigenous communities and understanding the microbiological profile of *masato* could contribute to establishing a public health response that is embedded in Indigenous knowledge and norms in Amazon Indigenous communities, especially where water potabilization is not yet implemented.

The aim of this research was to: (1) characterize key cultural features of *masato* with Shawi participants through participatory photography and (2) investigate the presence of total and fecal coliform bacteria in samples of *masato* compared to other communal water sources.

## Methods

This study is part of a larger, community-based research initiative focused on examining social and environmental drivers of health in remote Indigenous communities in Peru. The research methods and informed consent processes were approved by the Cayetano Heredia University Institutional Review Board, and the research ethics boards at McGill University, the University of Guelph, and the University of Alberta.

The research project took place in a Shawi Indigenous community located by the Armanayacu River, in the Balsa Puerto Amazonas District of the Loreto region in Peru.

During initial research in 2012, Shawi participants reported that food and water security were key health concerns related to recent social and environmental changes (Hofmeijer et al. 2013). Further, in 2014, Shawi participants used Photovoice to demonstrate the importance of *masato* as a key component of their daily diet (Zavaleta et al. 2018). Building on these previous results, we conducted microbiological testing of *masato* in November 2015 to explore its bacteriological properties and a follow-up photographic study in July 2017 to visually characterize practices, concepts, and key messages that participants chose to communicate about this traditional beverage.

### Photographic Study

Research has shown that photographic narratives are effective in promoting social change, balancing power, fostering trust, and recognizing ownership of Indigenous knowledge (Castleden et al. 2008; Harper et al. 2012; Lardeau et al. 2011). Beginning in 2012, the primary researchers (AB and CZC) have worked and lived within the community for a duration of 1–3 months annually. The photographer/researcher (MK) lived in the community for 2 months to establish a rapport and trust with community members. During the data collection in 2017, the photographer/researcher conducted informal, conversational interviews with community members and iteratively presented options for times and places for community members to be photographed, and to identify and prioritize what they wanted to communicate to others in Peru and abroad about masato. Photographs were classified into four main themes that represented the main food-related activities that community members practiced: eating, hunting, fishing, and farming. *Masato* emerged across all four themes, being the preferred way to quench thirst of Shawi participants. (To see further descriptions of this work and food-related themes, see Web site https://adaptandoparacomer.weebly.com/.) During community dissemination workshops in January 2018, Shawi community members validated the themes and final selection of photographs.

### Microbiological Study

In November 2015, a total of 37 water samples—19 household water samples, 11 *masato* samples, and 7 samples from outdoor drinking water sources (river, streams, and wells)—were collected over a 3-day period. We used purposive sampling to select households. Head of the households provided household water and *masato* samples in plastic or ceramic pots. Researcher then transferred the samples to sterile Whirl-Pak (Ziploc®) bags and placed them in a cooler at room temperature for transportation. For outdoor water sources, samples were taken far from the water’s edge and at medium depth, immersing the sampling bag into the direction of the current and approximately 15–20 cm below the water surface. Sites contaminated with garbage or close to sewage were avoided, as well as the collection of surface scum and sediment. Around 300 ml of liquid was collected from each drinking water source in a sterile Whirl-Pak bag and immediately put in a cooler for transportation.

Samples were processed in the community immediately after they were collected, within 20–30 min of collection. The working area was provided by the community—a room with no air current and no human or animal transit—and was intensely cleaned before sample manipulation. We used a membrane filter technique for detecting the presence of total and fecal coliform bacteria (American Public Health Association, American Water Works Association, & Water Environment Federation 1998). *Masato* samples were filtered using a sterile strainer to remove floating organic particles before dilution. Water and *masato* samples underwent serial dilutions with distilled water (two dilutions of 1:100); 100 ml of the last dilution was filtered through a cellulose filter membrane of pore size 0.22 μm and diameter 47 mm before placing them in Hach’s m-ColiBlue 24 Broth for cultivating. Inoculated plates (one per sample) were incubated at 35 °C for 24 h in a portable 12 V incubator connected to a car battery.

Total and fecal coliform counting was performed as presented in Hach’s Analytical Procedures protocol (Hach Company. Analytical Procedures. Coliforms: Membrane Filtration. 1999. USA). Chromogenic media discriminates *Escherichia coli* colonies as blue from other coliforms which show a red color.

## Results

### *Masato* Key Cultural Features

**Figure 1:**
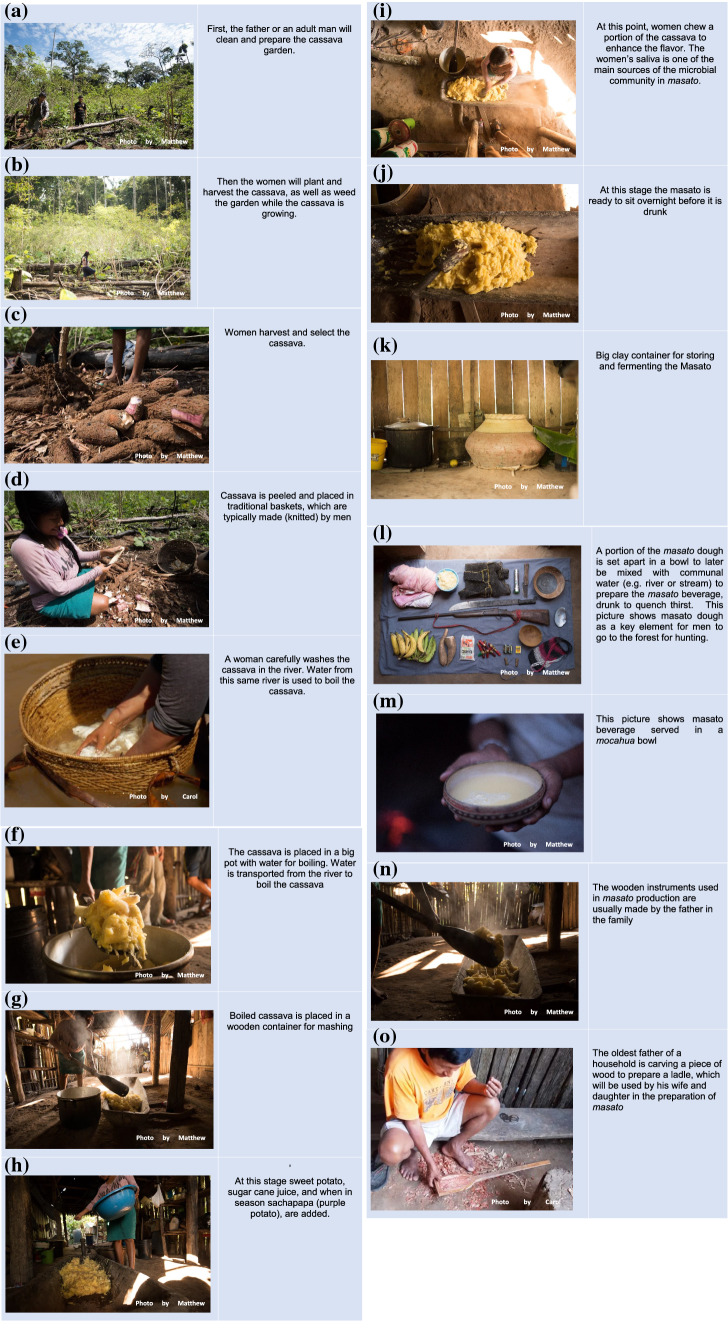
*Masato* preparation in one Shawi Indigenous community of the Peruvian Amazon. Shawi Indigenous people of the Peruvian Amazon transform cassava into a fermented beverage called *masato*. This series of pictures show the different stages of *masato* production: from planting cassava to preparing the beverage. It includes the cultural Shawi gender roles throughout the process. The typical preparation process is as follows: **a** The father or an adult male will initially clean and prepare the cassava garden; **b** then a women will plant and harvest the cassava, as well as weed the garden, while the cassava grows; **c** women harvest and select the cassava; **d** cassava is peeled and placed in traditional baskets that are typically made (knitted) by men; **e** cassava is carefully washed in the river by one woman, and water from this same river is used to boil the cassava; **f** cassava is placed in a big pot with water for boiling and water is transported from the river to boil the cassava; **g** boiled cassava is placed in a wooden container for mashing; **h** at this stage sweet potato, sugar cane juice, and, when in season, *sachapapa* (purple potato), are added; **i** at this point, women chew a portion of the cassava to enhance the flavor and the women’s saliva is one of the main sources of the microbial community present in *masato*; **j** then, the *masato* is ready to sit overnight before it is drank; **k** big clay containers are used for storing and fermenting the *Masato*; **l** a portion of the *masato* mash is set apart in a bowl to later be mixed with communal water (e.g., river or stream) for the preparation of the masato beverage, drunk to quench thirst, for men during hunting; **m** the masato beverage served in a mocahua bowl; **n** the wooden instruments used in cassava production are usually made by the father of the family; and, finally, **o** the oldest father of a household is carving a piece of wood to prepare a ladle, and his wife and daughter will use it in the preparation of *masato. Note* Pictures were taken during a photographic project in July 2017. All participants have provided their written consent to utilize pictures for academic and educative purposes.

*Masato* had cultural importance for Indigenous Shawi in the Peruvian Amazonia. Pictures in Fig. 1 illustrate how Shawi study participants transform cassava to drinkable *masato*: from cropping, cleaning and washing, cooking, mashing, resting for fermentation, to drinkable *masato*.

Along this process, there were a series of stages that were deeply rooted in Indigenous sociocultural practices and closely related to the benefits of natural ecosystems. For example, even though *masato* was exclusively prepared by women in all the study households, men also played a key role in the process. Men cleared the land for planting cassava (Fig. 1a) and fabricated wooden tools (Fig. 1n, o). Wood from the forest and mud from the river bed are used for making the utensils used in *masato* preparation. The fermentation of *masato* can take between 1 and 3 days, depending on preference, although some participants explained 1 day of fermentation may not be sufficient to produce *masato* that is safe to drink. A longer fermentation time is also preferred when the beverage is intended for consumption during a celebration. When ready for consumption, a portion of the fermented *masato* mash is combined with communal water sources to produce the drinkable version of *masato.* Figure 1l, m shows both stages of *masato*—the mash before being combined with water and the final drinkable beverage. In these Shawi communities, men were usually responsible for providing meat from the forest by hunting. When they go hunting, men reported taking *masato* mash with them to prepare *masato* along the road, as their source of drinking water (Fig. 1l).

### Coliform Presence in Masato

Coliform bacteria grew in all plates inoculated with water from household (19/19) and outdoor (7/7) sources. Colony-forming unit (CFU) counts for household water cultures varied from 1.4 × 10^3^ to 18.5 × 10^3^ to numbers too numerous to count (TNTC) per 1 ml (Table 1).

**Table 1:** Total and Fecal Coliform Count Per 1 ml from Water Samples Collected in a Shawi Community in the Amazon of Peru.

Sample	Source	Total coliform count	Fecal coliform count
1	Household (collected from the river)	TNTC	4.2 × 10^3^
2	Household (collected from the river)	TNTC	TNTC
3	Household (collected from the river)	TNTC	TNTC
4	Household (collected from the river)	TNTC	6 × 10^3^
5	Household (collected from the river)	TNTC	18.5 × 10^3^
6	Household (collected from the river)	TNTC	4.2 × 10^3^
7	Household (collected from the river)	TNTC	TNTC
8	Household (collected from the river)	TNTC	TNTC
9	Household (collected from the river)	TNTC	TNTC
10	Household (collected from the river)	TNTC	TNTC
11	Household (collected from the river)	TNTC	TNTC
12	Household (collected from the river)	TNTC	TNTC
13	Household (collected from the river)	TNTC	TNTC
14	Household (collected from a stream)	TNTC	TNTC
15	Household (collected from a stream)	TNTC	TNTC
16	Household (collected from a stream)	TNTC	18 × 10^3^
17	Household (collected from a well)	TNTC	2 × 10^3^
18	Household (collected from a well)	TNTC	1.4 × 10^3^
19	Household (collected from a well)	TNTC	8 × 10^3^
20	River	TNTC	1.6 × 10^3^
21	River	TNTC	TNTC
22	River	TNTC	TNTC
23	River	TNTC	TNTC
24	Stream	TNTC	TNTC
25	Stream	TNTC	17.2 × 10^3^
26	Well	TNTC	3.1 × 10^3^

*TNTC* too numerous to count.

Cultures from river, streams, and wells showed a higher load of coliform bacteria colonies than *masato* samples. Nine of eleven *masato* samples showed no bacterial growth (Table 2) (*P* < 0.001, Fisher’s exact test).

**Table 2:** Total and Fecal Coliform Count for 1 ml from *masato* Samples Collected in a Shawi Community in the Amazon of Peru.

Sample	Source	Total coliform count	Fecal coliform count
1	*Masato*	Negative	Negative
2	*Masato*	Confluent growth	Confluent growth
3	*Masato*	Negative	Negative
4	*Masato*	Negative	Negative
5	*Masato*	Negative	Negative
6	*Masato*	TNTC	TNTC
7	*Masato*	Negative	Negative
8	*Masato*	Negative	Negative
9	*Masato*	Negative	Negative
10	*Masato*	Negative	Negative
11	*Masato*	Negative	Negative

*TNTC* too numerous to count.

**Figure 2:**
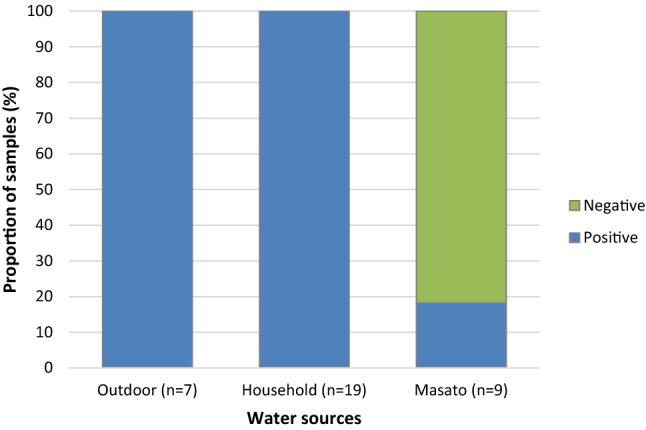
Presence of fecal coliform in water samples collected in a Shawi community in the Amazon of Peru.

Figure 1 shows the proportion of water samples with the presence of fecal coliform from each category: outdoor, household, and masato (Fig. 2).

## Discussion

A paradigm shift is needed in the public health community and health sector to consider and assess fermented Indigenous preparations, such as *masato,* as part of the local alternatives and technologies for treating contaminated water (Watson et al. 1996) and improving human health (Chilton et al. 2015). Our study demonstrates that coliform bacteria were significantly lower in *masato* compared with other sources of water in this location, suggesting additional health benefits of the beverage that have not previously been understood. This study is in agreement with a previous investigation of a similar fermented cassava beverage called “*chicha*” in Ecuador that also did not find fecal coliforms (Cox et al. 1987). Moreover, a recent study conducted among Indigenous people in the Amazon region of Bolivia suggested that water-rich foods, including cassava fermented beverage, represented a complex dietary adaptation to reduce exposure to pathogens from unsafe water (Rosinger and Tanner 2015).

### The Public Health Importance of *Masato*

Previous studies have reported on the nutritional importance of *masato* because it is made from locally available and locally produced cassava tubers (Creed-Kanashiro et al. 2009; Peña-Venegas et al. 2014). However, beyond its nutritional value, our findings suggest that *masato* is important because it is a culturally acceptable method to treating and drinking water in communities where potable water remains limited. Not only is it acceptable, *masato* preparation is embedded in Indigenous knowledge and also holds important cultural value for Indigenous people of the Amazon. As such, interest arises for its public health implications, embedded in Indigenous knowledge, for reducing the burden of waterborne diseases, especially in areas where access to clean water is not yet guaranteed.

Furthermore, our results raise the possibility that Indigenous masato preparation and consumption may be currently supporting a reduced burden of waterborne disease in Shawi and/or Amazonian communities. In 2007, a report from the Peruvian Ministry of Health found that for Shawi children between 1 and 19 years old, acute diarrhea was an important reason for consultation (Ormaeche Macassi 2008). Twenty-two percent of the youngest age-group (1–4 years old) had diarrhea (see “Appendix”); however, when we compared that rate with the results of the National Demographic and Family Health Survey for Peru in the same year 2007 (INEI 2009), the prevalence of acute diarrhea was 29% among children under 5 years old of Loreto Amazon region. Given that Shawi children drank masato, it suggests that fermented masato could have an implication on reducing acute bacterial diarrhea infections among young Shawi children.

The absence of coliform bacterial growth in most of the *masato* cultures in this study might be explained by the presence of alcohol and lactic acid product of the fermentation process involved in masato preparation. Motarjemi (2002), Şanlier et al. (2017), and O’Sullivan et al. (2002) indicate that the processes of fermentation—specifically lactic fermentation—inhibit the growth and survival of a variety of pathogenic organisms.

The high load of total and fecal coliforms found in the water sources of the community, other than *masato*, may be due to contamination of the main source of domestic water, the Armanayacu River. River water contamination stems from multiple factors, including open defecation by the river despite the availability of a few latrines in the community (Torres-Slimming et al. 2019). Additionally, wild and domestic animals (e.g., cattle and pigs) also roam river banks, adding to potential sources of water contamination. The high level of contamination in the communal water sources found in this study indicates that Shawi Indigenous communities are exposed to high risks if they would decide to drink water directly from natural sources, making the consumption of *masato* even more crucial while other public health solutions are being implemented.

### Future Research

In this study, all *masato* samples were provided by the head of the households, and handwashing and serving practices may have varied by household. Shawi have reported that they wash their hands before serving *masato*, although use of soap is not a common practice (Torres-Slimming et al. 2019). Future studies could analyze coliform load per preparation method, days of fermentation, and the effect of unwashed hands on coliform presence. Nonetheless, contamination post-fermentation could less of a problem with alcohol-containing beverages, as it will kill the pathogens on contact.

Future research should consider microbial testing beyond total and fecal coliform; analyzing *masato* samples along the multiple stages of its processing; determining practices that reduce the risk of contamination of *masato*; determining the mechanisms of microbiological clearing during the fermentation process (including the presence of microorganisms, ethanol, and lactic acid); and determining the direct or indirect effects of *masato* on the well-being of Indigenous people.

Furthermore, researchers have also suggested that beyond the health benefits of fermented Indigenous foods and beverages, these items could provide economic opportunities for improving Indigenous livelihoods (Marsh et al. 2014; Marshall and Mejia 2011). Future research assessing the economic role of *masato* in communities may be of value.

One limitation of our study was that we were not able to confirm the exact number of days of fermentation that each masato sample underwent. Variations in *masato* preparation methods were beyond the scope of this study; however, variations in preparation method may explain why 2 of the 11 *masato* samples showed coliform presence.

### The Importance of Culture and a Gender Approach to Promote Water Security

Our study found that *masato* preparation involved the participation of both men and women, suggesting that gendered cultural roles are also associated with the preparation and consumption of *masato.* It highlights the social function that this fermented beverage has for Indigenous peoples such as the Shawi in the Amazon region (González 2013). Our findings concur with previous studies that have reported on the importance, acceptability and the participation of women and men in producing masato among Shawi people from other communities (González 2013; Ormaeche and Valdez 2003).

Our results concur with research that posits that water treatment among remote Indigenous communities is not only a matter of implementing conventional forms of improving water quality (Jaravani et al. 2016; Wright et al. 2018). Clean water interventions need to consider local perceptions and cultural aspects, including the female and male responsibilities in assuring safe drinking water for family members and water utilization. For example, among some Indigenous communities in the global north, researchers reported that women perceive greater risks associated with the safety of drinking water than males, making female participation and knowledge key in designing water-related interventions (Dupont et al. 2014; Spence and Walters 2012). Moreover, designing culturally appropriate water treatment interventions that are embedded in Indigenous knowledge and practices avoids the colonial impacts that Indigenous populations, worldwide and in Peru, have experienced (Axelsson et al. 2016; Gracey and King 2009; Stephens et al. 2006).

## Conclusion

This study showed that *masato* plays an important role in reducing microbial contamination of untreated local water sources among Shawi participants. In addition, results show that there are Indigenous sociocultural implications related to this fermented beverage, including gender responsibilities during the preparation process. To warrant *masato* as a safe drinking water alternative, further investigation is needed on *masato* microbiological safety. Demonstrating the microbiological safety of *masato* could present important public health benefits for Indigenous communities where clean water cannot yet be guaranteed, as it is a culturally accepted drinking water alternative. To ensure the success of interventions aimed at improving access to drinking water, Indigenous community members must be active members of any intervention design.

## Data Availability

Pictures generated during the current study and informed consents are publicly available at the following address: https://adapttoeatperu.weebly.com/. Additional pictures are available from the corresponding author on reasonable request. All the microbiological data that support the findings in this study are already included in the article.

## Appendix

See Table 3.

**Table 3:** Description of the Most Frequent Pathologies that Explains up to the 80% of the Morbidity Among Shawi Children who Visited Local Health Service at the Balsa Puerto District Between Years 2002 and 2007. *Source*: Ormaeche Macassi (2008).

	Between 1 year old and 4 years old	Between 5 years old and 9 years old	Between 10 years old and 19 years old
	Total visits = 10,292	Total visits = 18,485	Total visits = 19,618
First	Other intestinal parasites (30%)	Other intestinal parasites (18%)	Malaria (17%)
Second	Other upper acute respiratory infections (29%)	Malaria (17%)	Iron deficiency anemia (15%)
Third	Diarrhea and other stomach conditions probably infections (22%)	Acute upper respiratory infections, acute pharyngitis and bronchitis and bronchiolitis (18%)	Other intestinal parasites (11%)
Fourth		Iron deficiency anemia (9%)	Other upper acute respiratory infection and acute pharyngitis (14%)
		Other types of physical trauma (6%)	Other types of physical trauma (7%)
Fifth		Skin and subcutaneous infections (6%)	Skin and subcutaneous infections (4%)
Sixth		Conjunctivitis and other conditions of the conjunctive (7%)	Other conditions of the urinary system (4%)
Seventh		Cavity (3%)	Conjunctivitis and other conditions of the conjunctive (2%)
Eight			Cavity (%2)
Ninth			Diarrhea and other stomach conditions, probably infections (2%)
Ten			Other anemias (2%)
